# Cardiovascular Magnetic Resonance in Early Detection of Radiation Associated Cardiotoxicity With Chest Radiation

**DOI:** 10.3389/fcvm.2022.867479

**Published:** 2022-05-31

**Authors:** Srilakshmi Vallabhaneni, Yue Wang, Ying Zhang, Amanda Smith, Wei Zou, Steven Feigenberg, John Plastaras, Gary Freedman, Walter R. T. Witschey, Bonnie Ky, Yuchi Han

**Affiliations:** ^1^Cardiovascular Division, Department of Medicine, University of Texas Southwestern Medical Center, Dallas, TX, United States; ^2^Cardiovascular Division, Department of Medicine, Perelman School of Medicine of the University of Pennsylvania, Philadelphia, PA, United States; ^3^Department of Cardiology, Shanghai Ninth People’s Hospital, Shanghai Jiao Tong University School of Medicine, Shanghai, China; ^4^Department of Cardiology, People’s Liberation Army (PLA) General Hospital, Beijing, China; ^5^Department of Radiation Oncology, Perelman School of Medicine of the University of Pennsylvania, Philadelphia, PA, United States; ^6^Department of Radiology, Perelman School of Medicine of the University of Pennsylvania, Philadelphia, PA, United States; ^7^Cardiovascular Division, Department of Internal Medicine, Davis Heart and Lung Research Institute, The Ohio State University Wexner Medical Center, Columbus, OH, United States

**Keywords:** chemotherapy associated cardiotoxicity, radiation cardiotoxicity, cardiooncology, prospective cohort study, CMR

## Abstract

**Background:**

Chest radiation therapy (RT) is known to be associated with cardiotoxicity. However, the changes in myocardial tissue characterization with radiation-induced cardiotoxicity are not well-understood.

**Objectives:**

This study sought to assess the changes in left ventricular function and tissue characterization using cardiovascular magnetic resonance (CMR) in patients receiving RT.

**Materials and Methods:**

Between June 2015 and July 2018, we enrolled patients with breast, lung cancer, or lymphoma with plan to receive chest radiation after chemotherapy. CMR was performed using a 1.5T scanner at baseline and 6 months after RT. Myocardial volume, function, strain analysis using feature tracking, and tissue characterization including late gadolinium enhancement (LGE), T1, T2, T1ρ (rho), and extracellular volume fraction (ECV) were measured and compared using non-parametric methods.

**Results:**

The final cohort consisted of 16 patients, 11 of whom completed both baseline and follow-up CMRs. Patients were matched to 10 healthy controls. At baseline prior to RT, compared to controls, patients had lower global circumferential strain (GCS) (15.3 ± 2.2% vs.18.4 ± 2.1%, *p* = 0.004), and elevated T2 (47.9 ± 4.8 ms vs. 45.0 ± 1.5 ms, *p* = 0.04) and T1ρ values (78.4 ± 5.9 vs. 66.9 ± 4.6 ms, *p* < 0.001). Two patients had LGE. There was no significant difference in the average T1 values or ECV. There was a trend toward lower LV ejection fraction and global longitudinal strain (GLS). At 6-month follow-up after RT, there were no significant changes in all the CMR parameters.

**Conclusion:**

At 6-month following chest radiation therapy, there was no change in LV and RV EF, LV and RV GLS, LV GCS, and myocardial tissue characterization using LGE, T1, ECV, T2, and T1ρ in a small cohort of patients. However, the baseline T2 and T1ρ were elevated and LV GCS was reduced compared to controls indicating ongoing myocardial edema and subclinical dysfunction post-chemotherapy.

## Introduction

As survival continues to improve following treatment for cancer, there is an increasing concern for long-term treatment-related toxicity. In the management of thoracic malignancies, systemic therapy and radiation are associated with cardiotoxicity. Radiation therapy (RT) is an important component of curative treatment in about half of patients with cancer (1). However, thoracic RT may be associated with a significant risk of cardiovascular (CV) toxicity and is of great concern, particularly in breast and lung cancer patients (2, 3). Ischemic heart disease, valvular disease, non-ischemic cardiomyopathy, pericardial disease, arrhythmias, and conduction abnormalities are each potential adverse cardiovascular sequelae of chest radiation (4–7). Despite improvements in RT techniques and modalities that decrease the total radiation dose administered, radiation-induced heart disease (RIHD) remains a common cause of morbidity and mortality among cancer survivors. The impact of cardiotoxicity with current RT techniques on cardiac function, cardiomyocyte inflammation, and necrosis is unknown (8).

The etiology of RIHD is not well-understood (9) although multiple pathophysiologic mechanisms exist for long-term RIHD, including micro- and macrovascular injury, fibrosis, and endothelial cell dysfunction (10, 11). Currently, available biomarkers such as troponin and brain natriuretic peptide have not been validated in predicting the development of RIHD (12–14). These biomarkers are known to play an important role in the prediction of cardiotoxicity in patients receiving cardiotoxic cancer therapy, particularly with sequential anthracycline and trastuzumab therapy (15). Multiple cardiac imaging modalities play important roles in pretreatment risk assessment, early detection of cardiotoxicity, and in the identification of long-term cardiotoxicity after receiving potentially cardiotoxic treatment. Cardiovascular magnetic resonance imaging (CMR) provides an accurate assessment of cardiac volumes, systolic function and can non-invasively characterize myocardial tissue and allow early detection of subclinical cardiac injury (16).

Cardiovascular magnetic resonance utilizes a multiparametric approach for myocardial evaluation. Left ventricular (LV), global longitudinal strain (GLS), and circumferential strain (GCS) by CMR feature tracking is a more sensitive measure of systolic function and can be used to identify sub-clinical LV dysfunction (17). Elevated native T1 values correlate with fibrosis or increased interstitial expansion (18). Native and post-contrast T1 mapping are used to calculate myocardial extracellular volume (ECV) which correlates with extracellular volume fraction and its elevation maybe a result of fibrosis, edema, protein deposition, or other pathologic processes (19). Myocardial edema can be assessed quantitatively with T2 mapping. T1ρ or spin-locking relaxation time is a promising non-contrast sequence that is sensitive to edema and necrosis in the acute setting and scar in chronic myocardial infarction (20). We sought to study the myocardial changes (volume, function, strain, fibrosis/edema) with CMR in patients undergoing RT after chemotherapy.

## Materials and Methods

### Study Design and Patient Eligibility

Patients with breast cancer, lung cancer, or lymphoma receiving chest radiation between June 2015 and July 2018 at our institution were considered eligible for this sub-study (21). Patients were excluded if they had documented prior cardiac disease history including heart failure, uncontrolled hypertension, coronary artery disease, or arrhythmias. All patients received RT to the cancer-specific tumor region to a curative dose appropriate for their specific tumor per institutional guidelines. The dose to the heart and the dose to the left ventricle were recorded. Control subjects were healthy volunteers without any known cardiovascular or pulmonary disease who were not taking any regular medications. The study was approved by the institutional review board at our university. Written informed consent was obtained from all subjects in the study.

### Cardiovascular Magnetic Resonance

Cardiovascular magnetic resonance was performed before RT and at 6 months after RT using a 1.5 scanner (Avanto, Siemens, Germany). For patients with breast cancer and lymphoma, chemotherapy was administered before baseline CMR, while the lung cancer patients received chemotherapy during RT. Retrospectively gated cine images of the short axis of the heart were obtained using steady-state free precession (SSFP) for biventricular volumes and function. Sequence parameters include TE (echo time)/TR (repetition time) = 1.07/2.2 ms; slice thickness = 8 mm with 2 mm gap; bandwidth = 930 Hz/pixel; flip angle = 59°; field-of-view = 320-380 mm^2^; spatial resolution = 1.97 × 1.97 mm^2^, parallel imaging factor = 2. Late gadolinium imaging was obtained using routine 2D inversion recovery sequences with the following parameters: TE = 3.3 ms; slice thickness = 8 mm with 2 mm gap; bandwidth = 130 Hz/pixel; flip angle = 15, field-of-view = 320–380 mm; spatial resolution = 1.5 × 1.5 mm.

All mapping sequences were obtained at the basal and mid ventricular short axis. Native T1 maps were acquired using a modified Looker-Locker Inversion Recovery (MOLLI) sequence (Siemens Work-in-Progress 448B) using the 5(3)3 scheme before and 4(1)3(1)2 scheme after contrast injection in diastole with the following sequence parameters: TE = 1.03 ms; minimum TI = 100 ms; TI increment = 80 ms; flip angle = 35°, field-of-view = 320–380 mm^2^; spatial resolution = 1.7 × 1.7 mm^2^; slice thickness = 8 mm; bandwidth = 1,085 Hz/pixel; parallel imaging factor = 2. T2 maps were acquired using a T2-prepared SSFP sequence with the following sequence parameters at end-systole: T2-preparation durations = 0, 24, and 55 ms; TE/TR = 1.37/2.64 ms; flip angle = 70°; field-of-view = 320 × 380 mm^2^; bandwidth = 1,185 Hz/pixel, spatial resolution = 1.9 × 2.3 mm, slice thickness = 8 mm; parallel imaging factor = 2. Motion corrected T1ρ maps were acquired at the same location and eight T1ρ weighted images with different spin locking pulse duration (TSL) was performed: TSL = 2, 10, 18, 26, 34, 42, 50 ms, B1 = 400–500 Hz, spatial resolution = 1.4 × 1.4 mm^2^, slice thickness = 6 mm, flip angle = 70°, TE = 1.45 ms, TR = 2.9 ms, bandwidth = 900 Hz/pixel, linear k-space phase encoding ordering, parallel imaging with acceleration factor = 2.

### Image Analysis

Left ventricular and right ventricular (RV) endocardial and epicardial borders were manually traced on short-axis slices according to Society of Cardiovascular Magnetic Resonance (SCMR) recommendations using Qmass software (Medis, Netherlands) (22). Papillary muscles were included in the ventricular volume. To discriminate ventricular volume from the atrial volume at the basal slices, four-chamber view was used as a reference. Strain analyses were analyzed on long-axis and short-axis cine images using SuiteHeart (Neosoft, WI). Mean values from T1, T2, and T1ρ mapping slices were obtained by drawing a region of interest on the reconstructed maps over the septal and lateral wall avoiding the blood pool and epicardial fat. The mean mapping values were then averaged between the basal and the mid-ventricular slices. Mapping values are obtained on AQNetClient (Durham, NC, United States). LGE is assessed visually by an experienced investigator with 15-year CMR experience.

### Statistical Analysis

Continuous variables were expressed as mean ± SD. Categorical variables were presented as absolute numbers or percentages. For continuous variables, significant differences were analyzed using the Mann-Whitney *U*-test between the two groups and Kruskal–Wallis H (K) test among the three groups. For ranked variables, significant differences were analyzed using the Chi-square test. A *p*-value of < 0.05 was considered statistically significant. Data analyses were performed using the SPSS 17.0 (SPSS Inc., Chicago, IL, United States).

## Results

**FIGURE 1:**
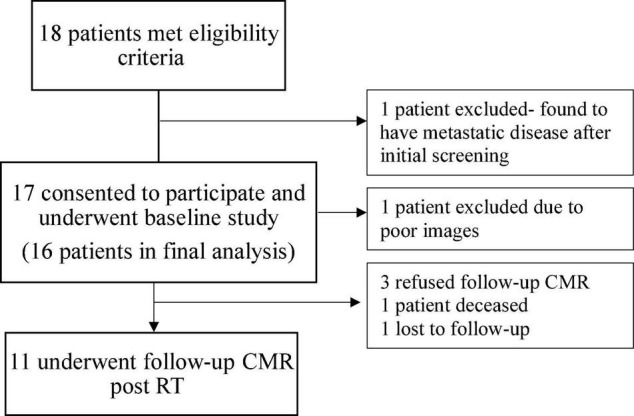
Diagram illustrating the selection of participants for the study. CMR, cardiovascular magnetic resonance; RT, radiation therapy.

A total of 18 patients met eligibility criteria and underwent baseline CMR (Figure 1). One patient was excluded due to diagnosis of metastatic disease after enrollment and one patient due to suboptimal baseline imaging. The final cohort included 16 patients of which 11 patients underwent follow-up CMR 6 months after completion of RT.

Of the 16 patients, 10 patients underwent treatment with anthracycline (AC) based chemotherapy and six patients with non-AC based chemotherapy. Five patients received chemotherapy (all were non-AC-based) during radiation. At baseline, when compared to controls, patients who underwent RT had reduced LV GCS at baseline (15.3 ± 2.2% vs. 18.4 ± 2.1%, *p* = 0.004) and a trend toward lower LV ejection fraction (EF) (56.3 ± 6.6% vs. 59.7 ± 2.8%, *p* = NS). The T2 and T1ρ values were elevated in these patients compared with controls at baseline (47.9 ± 4.8 ms vs. 45.0 ± 1.5 ms, *p* = 0.04 and 78.4 ± 5.9 ms vs. 66.9 ± 4.6 ms, *p* < 0.001). No significant difference in the average T1 values was observed in these patients (1018.4 ± 30.7 ms) compared to the control group (998.0 ± 22.9 ms, *p* = NS). No significant differences were found in the right ventricle for ventricular volumes, function, and RV longitudinal strain (Table 1).

**TABLE 1:** Subject baseline characteristics.

Variables	Control (*n* = 10)	Patients (*n* = 16)	*p*-Value
Age	42.0 ± 13.5	47.3 ± 17.8	0.37
Male (%)	40	43.8	0.854
BSA	1.9 ± 0.3	2.0 ± 0.3	0.445
**CMR characteristics**			
Peak LV GLS (%)	20.2 ± 2.5	18.4 ± 3.2	0.061
Peak LV GCS (%)	18.4 ± 2.1	15.3 ± 2.2	**0.004**
LVEDVi (mL/m^2^)	81.3 ± 12.8	85.5 ± 15.4	0.527
LVEF (%)	59.7 ± 2.8	56.3 ± 6.6	0.114
LVmassi (g/m^2^)	45.1 ± 9.4	47.5 ± 9.3	0.673
Peak RV GLS (%)	23.9 ± 3.2	22.2 ± 4.3	0.414
RVEDVi (mL/m^2^)	90.1 ± 17.5	81.6 ± 17.1	0.343
RVEF (%)	53.8 ± 4.2	56.4 ± 4.6	0.206
LGE (+)	/	2 (17)	/
T1 (ms)	998.0 ± 22.9	1018.4 ± 30.7	0.082
T2 (ms)	45.0 ± 1.5	47.9 ± 4.8	**0.04**
T1ρ (ms)	66.9 ± 4.6	78.4 ± 5.9	**<0.001**
ECV	24.9 ± 2.8	21.2 ± 10.9	0.958

*BSA, body surface area; CMR, cardiovascular magnetic resonance; ECV, extracellular volume; GCS, global circumferential strain; GLS, global longitudinal strain; LGE, late gadolinium enhancement; LV, left ventricle; LVEF, left ventricular ejection fraction; LVEDVi, left ventricular end-diastolic volume indexed; RV, right ventricle; RVEF, right ventricular ejection fraction; RVEDVi, right ventricular end-diastolic volume indexed. Bold p-values are statistically significant.*

**FIGURE 2:**
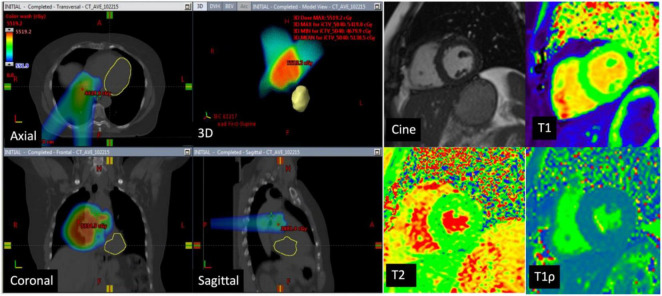
CT images in a 73-year-old man with lung cancer—Axial, coronal, sagittal, and 3D CT images with color-washed dose distribution. The heart is depicted in yellow and is not in the planned radiation field. The patient received a total of 50.4 Gy of which the mean dose to the left ventricle was 0.02 Gy. Cardiac magnetic resonance (CMR) images at baseline with normal LVEF (65%), normal native T1 mapping (1,043 ms), normal T2 mapping (47 ms), and elevated T1ρ at 78 ms. Follow-up CMR in 6 months showed no significant difference in native T1 and T2 values and the T1ρ values remained elevated.

**FIGURE 3:**
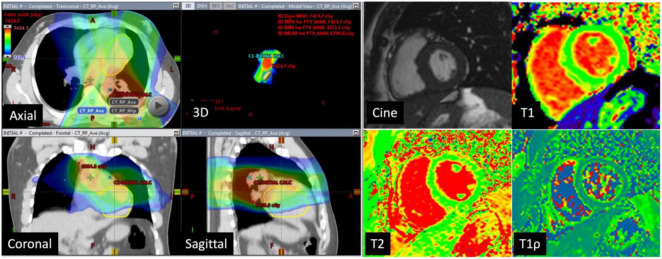
CT images in a 73-year-old man with lung cancer: Axial, coronal, sagittal, and 3D CT images with color-washed dose distribution. The heart depicted in the yellow is in the radiation field. The patient received a total of 66.6 Gy of which the mean dose to the left ventricle was 31.6 Gy. Cardiac magnetic resonance (CMR) images at baseline with normal LVEF (60%), normal native T1 mapping (983 ms), normal T2 mapping (43 ms), and elevated T1ρ at 87 ms.

Eleven of the 16 patients completed follow-up CMR. The mean age was 48.3 ± 18.6 years and 55% were female. Contemporary radiation techniques to minimize radiation to the heart while ensuring full dose to the tumor were used. These include intensity-modulated radiation therapy (IMRT), 3-dimensional conformal RT (3DCRT) in 25 and 44% of the patients, respectively. 3D CRT was exclusively used in the setting of breast cancer and lymphoma. Proton therapy was used in 31% of the patients with either passive scattering (12% of the patients) which produces a broad beam of proton delivery or with active scanning (19% of the patients) which uses several small pencil beams throughout the target volume (Table 2). The average radiation dosage was 45.1 ± 15.7 Gy. The maximum and mean heart dose of this cohort were averaged at 38.2 Gy (range 21.6–69.1 Gy) and 6.45 Gy (range 0.6–30.7 Gy). Of these, seven patients received a mean heart dose of less than 5 Gy, two patients between 5 and 10 Gy and one patient at a dose greater than 10 Gy and one patient at a dose greater than 20 Gy. The maximum and mean dose to the LV of this cohort averaged 26.6 Gy (range 0.2–67.8 Gy) and 5.5 Gy (range 0–31.6 Gy). Patients were then divided into those who received minimal radiation and partial/full radiation. Patients who received minimal radiation are breast cancer patients with radiation primarily to the breast and right side of the heart in the radiation field (Figure 2). The partial/full radiation patients had LV in the radiation field (Figure 3). There was no significant difference in LV or RV EF, LV or RV volumes, strain (LV GLS, LV GCS, and RV GLS), T1, T2, and T1ρ values after RT compared to pre-RT in these two cohorts (Table 3). Two patients had positive LGE which did not change after RT.

**TABLE 2:** Cancer and radiation characteristics of patients.

Variables	Patients (*n* = 16)
Age (years)	47.25 ± 17.8
Male (%)	7 (67)
BSA (m^2^)	1.98 ± 0.28
**Type of cancer**	
Breast cancer	6 (38%)
Lung cancer	5 (31%)
Hodgkin lymphoma	5 (31%)
**Cardiac risk factors**	
Hypertension	4 (25)
Type II diabetes	2 (12.5)
Hyperlipidemia	6 (37.5)
**Radiation treatment type**	
3D Conformal	7 (44%)
Protons (passive scattering)	2 (12%)
Protons (scanning)	3 (19%)
IMRT	4 (25%)
Total radiation dose (Gy)	45.1 ± 15.7
**RT dose to heart**	
Mean dose (Gy)	6.5 ± 8.6
Maximum dose (Gy)	38.2 ± 14.4
**RT dose to left ventricle**	
Mean dose (Gy)	5.5 ± 9.2
Maximum dose (Gy)	26.6 ± 21.6

*BSA, body surface area; 3D, three-dimensional; IMRT, intensity-modulated radiation therapy; RT, radiation therapy.*

**TABLE 3:** Cardiovascular magnetic resonance (CMR) characteristics pre- and post-RT in different radiation doses to the heart and left ventricle.

Variables	Absolute change		*p*-Value	% Change		*p*-Value
			
	Patients with minimal radiation (*n* = 6)	Patients with partial/full radiation (*n* = 5)		Patients with minimal radiation (*n* = 6)	Patients with partial/full radiation (*n* = 5)	
Peak LV GLS (%)	–1.3 ± 0.7	–3.6 ± 3.7	0.144	–6.8 ± 3.1	–15.2 ± 15.2	1
Peak LV GCS (%)	–0.6 ± 1.0	–1.4 ± 2.4	0.583	–4.0 ± 6.9	–8.4 ± 14.5	0.584
LVEDVi (mL/m^2^)	0.6 ± 7.4	–5.3 ± 10.2	0.465	0.7 ± 8.4	–7.8 ± 15.8	0.465
LVEF (%)	–5.1 ± 4.8	–6.2 ± 4.4	0.465	–8.9 ± 8.6	–10.4 ± 7.7	0.584
LVmassi (g/m^2^)	1.0 ± 1.9	–0.5 ± 2.7	0.361	2.3 ± 4.0	–1.8 ± 7.5	0.361
Peak RV GLS (%)	2.5 ± 4.3	2.7 ± 5.6	0.715	13.0 ± 22.1	18.4 ± 36.6	0.584
RVEDVi (mL/m^2^)	–4.5 ± 7.5	–7.7 ± 13.0	1	–5.7 ± 10.2	–11.2 ± 19.0	1
RVEF (%)	–1.3 ± 1.8	–1.7 ± 4.8	0.584	–2.5 ± 3.7	–3.1 ± 9.0	0.584
T1 (ms)	–38.6 ± 21.7	–14.1 ± 37.9	0.273	–3.7 ± 2.0	–1.3 ± 3.7	0.273
T2 (ms)	–2.4 ± 7.5	1.7 ± 4.3	0.361	–3.4 ± 12.1	3.9 ± 9.5	0.273
T1ρ (ms)	–3.9 ± 5.4	0.8 ± 11.4	0.67	–4.7 ± 6.6	1.0 ± 14.6	0.67
ECV (%)	–3.5% ± 5.9%	–2.1% ± 0.7%	0.513	–11.5 ± 20.8	–8.1 ± 2.9	0.513

*CMR, cardiovascular magnetic resonance; ECV, extracellular volume; GCS, global circumferential strain; GLS, global longitudinal strain; LGE, late gadolinium enhancement; LV, left ventricle; LVEF, left ventricular ejection fraction; LVEDVi, left ventricular end-diastolic volume indexed; RT, radiation therapy; RV, right ventricle; RVEF, right ventricular ejection fraction; RVEDVi, right ventricular end-diastolic volume indexed.*

## Reproducibility

There was excellent intra and inter-observer reproducibility with the ICC ranging from 0.892–0.964 for T1, T2, and T1ρ values (Table 4).

**TABLE 4:** The interclass correlation and intraclass correlation of tissue characterization.

*c*	Intraclass correlation	*P*	Interclass correlation	*P*
T1	0.922	<0.001	0.938	<0.001
T2	0.892	<0.001	0.896	<0.001
T1ρ	0.964	<0.001	0.929	<0.001

## Discussion

We prospectively evaluated for early cardiac toxicity following RT using CMR to identify subclinical functional and structural changes. Prior to chest RT, we have found reduced GCS, elevated T2 and T1ρ values in patients compared to controls indicating myocardial edema and subclinical functional changes after chemotherapy. After RT, a 6-month follow-up CMR showed no change in LV and RV EF, LV and RV GLS, LV GCS, T1/ECV, T2, and T1ρ values.

All patients in this study received chemotherapy prior to RT and 11/17 patients received anthracycline-based chemotherapy. When compared to controls, patients who underwent chemotherapy had elevated T2 and T1ρ values at baseline, which were possibly related to chemotherapy and active inflammation/fibrosis (23). Both T2 and T1ρ mapping are sensitive to myocardial edema with T1ρ also sensitive to fibrotic changes. T1ρ is a novel imaging parameter and has been shown to be elevated in myocardial infarction and hypertrophic cardiomyopathy (20, 24). These findings along with reduced LV GCS indicate the myocardial effect of chemotherapy prior to initiation of RT. The SUCCOUR (Strain Surveillance of Chemotherapy for Improving Cardiovascular Outcomes) trial, an international multicenter prospective randomized controlled trial, showed that changes in echocardiographic GLS in patients undergoing cardiotoxic chemotherapy predict incident LV systolic dysfunction (25). In our study, LV GLS trended toward significance, but LV GCS was more sensitive to detect subclinical LV dysfunction after chemotherapy using CMR. However, neither GCS nor GLS was further reduced in 6 months after RT in our cohort. These strain markers for subclinical functional changes and myocardial tissue characterization showed no significant change in the short-term with RT, but the long-term effect of these changes would need longer studies to elucidate.

A few studies in patients receiving anthracycline-based chemotherapy have shown elevated T1 and ECV which can occur as early as 3 months after initiation of therapy (26–28). However, other studies have shown an acute decrease in T1 values 48 h after anthracycline administration (29). A porcine model of doxorubicin toxicity showed an early increase in T1 values (2 weeks after three doses of doxorubicin) with unchanged T2 and ECV values (30). Takagi et al. (31) prospectively assessed changes in LV volumes, native T1, and ECV in 24 patients with esophageal cancer receiving RT with CMR at baseline, 6-month, and 1.5 years using a 3 Tesla MRI scanner. Their study showed early myocardial changes with elevated native T1 and ECV in the septum at 6-month compared to baseline (1257.0 ± 6.4 ms vs. 1183.0 ± 6.5 ms; 32.0 ± 6.3% vs. 26.0 ± 6.3%; *p* < 0.01 for both) and LV stroke volume index reduced and more patients had LGE at 1.5 years compared to baseline (36 ± 9 mL/m^2^ vs. 41 ± 11 mL/m^2^; *p* = 0.046; 78% [11/14] vs. 7% [1/14]; *p* < 0.01) (31). The mean radiation dose to the septum and apical lateral wall was 43 ± 4 Gy and 3 ± 4 Gy respectively which is higher compared to our cohort. Patients in our study cohort underwent CMR 6 months post-RT which did not show an elevation in native T1/ECV compared to baseline. This difference might be explained by the newer radiation techniques with lower mean radiation dose to the heart and the lack of esophageal cancer patients who tend to receive higher cardiac doses.

Several other studies utilized LGE/T1 sequences to detect fibrosis post-RT. Ricco et al. (32) studied 28 patients with chest tumors who underwent CMR on average 46.4 months post-RT on a 1.5 Tesla MRI scanner. The average heart dose in this cohort was 50.9 Gy (maximum heart dose) and 8.2 Gy (mean heart dose). LGE was present in 9 (32%) patients and there was no significant difference in cardiac radiation doses between patients who had LGE and those who did not. The average T1 value of the LV septum in this study was 1,009 ms (range 933–1,117 ms), similar to our study. However, patients in this study did not have a baseline CMR, so it is unclear if LGE developed post-RT (32). Another study by Umezawa et al. (33) evaluated 24 esophageal cancer patients who were treated with concurrent radiation therapy and found that 50% of the patients had LGE at a median time of 23.5 months to CMR after completion of RT. The LGE was present only within the segments of 40 Gy or 60 Gy isodose distribution (33). Another study by Tuohinen et al. (34) in 20 left breast cancer patients on a 3 Tesla MRI scanner showed elevated T1 values of 6 years after RT within the inferoseptal walls of the LV with mean heart dose being a predictor of elevated T1 values. All these studies examined the cardiac effects of RT later than our study.

The time to development of fibrosis with RT is not well-known but based on previous studies and our current study, it does not seem to develop until after 6 months of radiation therapy and is heart-dose dependent. Additional tissue characterization markers such as T2 and T1ρ may indicate ongoing myocardial edema that was a consequence of chemotherapy, but the long-term implications of these findings will need to be studied in the future.

## Limitations

Our study has several limitations. First, our sample size is small with a few different types of cancers/chemotherapy regimens and radiation fields. Second, patients did not have baseline CMR prior to chemotherapy. The mean cardiac dose in our cohort is low considering that less than 5% of lung cancer patients have mean cardiac doses below 10 Gy. To further study myocardial tissue changes in RT, a larger population study is needed.

## Conclusion

Six-month follow-up CMR in patients undergoing chest radiation therapy showed no change in LV and RV EF, LV and RV GLS, LV GCS, LGE, T1/ECV, T2, and T1ρ values. Prior to chest RT, we found reduced GCS, and elevated T2 and T1ρ values in patients after chemotherapy as compared to controls. Contemporary chest radiation with a low heart dose may not have short-term effects on the heart although future studies are needed to confirm these findings.

## Data Availability Statement

The original contributions presented in the study are included in the article/supplementary material, further inquiries can be directed to the corresponding author.

## Ethics Statement

The studies involving human participants were reviewed and approved by IRB at University of Pennsylvania. The patients/participants provided their written informed consent to participate in this study.

## Author Contributions

All authors participated in the writing and/or final review and approval of this manuscript.

## Conflict of Interest

The authors declare that the research was conducted in the absence of any commercial or financial relationships that could be construed as a potential conflict of interest.

## Publisher’s Note

All claims expressed in this article are solely those of the authors and do not necessarily represent those of their affiliated organizations, or those of the publisher, the editors and the reviewers. Any product that may be evaluated in this article, or claim that may be made by its manufacturer, is not guaranteed or endorsed by the publisher.

## References

[1] Delaney G Jacob S Featherstone C Barton M. The role of radiotherapy in cancer treatment: estimating optimal utilization from a review of evidence-based clinical guidelines. *Cancer.* (2005) 104:1129–37. 10.1002/cncr.21324 16080176

[2] Darby SC Ewertz M McGale P Bennet AM Blom-Goldman U Brønnum D Risk of ischemic heart disease in women after radiotherapy for breast cancer. *N Engl J Med.* (2013) 368:987–98. 10.1056/NEJMoa1209825 23484825

[3] Zamorano JL Lancellotti P Rodriguez Muñoz D Aboyans V Asteggiano R Galderisi M 2016 ESC position paper on cancer treatments and cardiovascular toxicity developed under the auspices of the ESC committee for practice guidelines: the task force for cancer treatments and cardiovascular toxicity of the European society of cardiology (ESC). *Eur J Heart Fail.* (2017) 19:9–42. 10.1002/ejhf.654 27565769

[4] Hooning MJ Botma A Aleman BM Baaijens MH Bartelink H Klijn JG Long-term risk of cardiovascular disease in 10-year survivors of breast cancer. *J Natl Cancer Inst.* (2007) 99:365–75. 10.1093/jnci/djk064 17341728

[5] van Nimwegen FA Schaapveld M Janus CP Krol AD Petersen EJ Raemaekers JM Cardiovascular disease after Hodgkin lymphoma treatment: 40-year disease risk. *JAMA Intern Med.* (2015) 175:1007–17. 10.1001/jamainternmed.2015.1180 25915855

[6] Hancock SL Tucker MA Hoppe RT. Factors affecting late mortality from heart disease after treatment of Hodgkin’s disease. *JAMA.* (1993) 270:1949–55. 8411552

[7] Veinot JP Edwards WD. Pathology of radiation-induced heart disease: a surgical and autopsy study of 27 cases. *Hum Pathol.* (1996) 27:766–73. 10.1016/s0046-8177(96)90447-5 8760008

[8] Machann W Beer M Breunig M Störk S Angermann C Seufert I Cardiac magnetic resonance imaging findings in 20-year survivors of mediastinal radiotherapy for Hodgkin’s disease. *Int J Radiat Oncol Biol Phys.* (2011) 79:1117–23. 10.1016/j.ijrobp.2009.12.054 20385449

[9] Cahlon O Khan AJ. Cardiac toxicity: the more we learn, the less we know. *Int J Radiat Oncol Biol Phys.* (2017) 99:1162–5. 10.1016/j.ijrobp.2017.08.048 29165284

[10] Schultz-Hector S Trott KR. Radiation-induced cardiovascular diseases: is the epidemiologic evidence compatible with the radiobiologic data? *Int J Radiat Oncol Biol Phys.* (2007) 67:10–8. 10.1016/j.ijrobp.2006.08.071 17189062

[11] Wu W Masri A Popovic ZB Smedira NG Lytle BW Marwick TH Long-term survival of patients with radiation heart disease undergoing cardiac surgery: a cohort study. *Circulation.* (2013) 127:1476–85. 10.1161/CIRCULATIONAHA.113.001435 23569119

[12] Lancellotti P Nkomo VT Badano LP Bergler-Klein J Bogaert J Davin L Expert consensus for multi-modality imaging evaluation of cardiovascular complications of radiotherapy in adults: a report from the European association of cardiovascular imaging and the American society of echocardiography. *J Am Soc Echocardiogr.* (2013) 26:1013–32. 10.1016/j.echo.2013.07.005 23998694

[13] Demissei BG Freedman G Feigenberg SJ Plastaras JP Maity A Smith AM Early changes in cardiovascular biomarkers with contemporary thoracic radiation therapy for breast cancer, lung cancer, and lymphoma. *Int J Radiat Oncol Biol Phys.* (2019) 103:851–60. 10.1016/j.ijrobp.2018.11.013 30445173 PMC6722323

[14] Gomez DR Yusuf SW Munsell MF Welsh JW Liao Z Lin SH Prospective exploratory analysis of cardiac biomarkers and electrocardiogram abnormalities in patients receiving thoracic radiation therapy with high-dose heart exposure. *J Thorac Oncol.* (2014) 9:1554–60. 10.1097/JTO.0000000000000306 25521400 PMC4273574

[15] Demissei BG Freedman G Feigenberg SJ Plastaras JP Maity A Smith AM Early changes in cardiovascular biomarkers with contemporary thoracic radiation therapy for breast cancer, lung cancer, and lymphoma. *Int J Radiat Oncol Biol Phys.* (2019) 103:851–60. 10.1016/j.ijrobp.2018.11.013 30445173 PMC6722323

[16] Thavendiranathan P Wintersperger BJ Flamm SD Marwick TH. Cardiac MRI in the assessment of cardiac injury and toxicity from cancer chemotherapy: a systematic review. *Circ Cardiovasc Imaging.* (2013) 6:1080–91. 10.1161/CIRCIMAGING.113.000899 24254478

[17] Onishi T Saha SK Delgado-Montero A Ludwig DR Onishi T Schelbert EB Global longitudinal strain and global circumferential strain by speckle-tracking echocardiography and feature-tracking cardiac magnetic resonance imaging: comparison with left ventricular ejection fraction. *J Am Soc Echocardiogr.* (2015) 28:587–96. 10.1016/j.echo.2014.11.018 25577185

[18] Ugander M Bagi PS Oki AJ Chen B Hsu LY Aletras AH Myocardial edema as detected by pre-contrast T1 and T2 CMR delineates area at risk associated with acute myocardial infarction. *JACC Cardiovasc Imaging.* (2012) 5:596–603. 10.1016/j.jcmg.2012.01.016 22698528 PMC3769169

[19] Miller CA Naish JH Bishop P Coutts G Clark D Zhao S Comprehensive validation of cardiovascular magnetic resonance techniques for the assessment of myocardial extracellular volume. *Circ Cardiovasc Imaging.* (2013) 6:373–83. 10.1161/CIRCIMAGING.112.000192 23553570

[20] Han Y Liimatainen T Gorman RC Witschey WR. Assessing myocardial disease using T_1ρ_ MRI. *Curr Cardiovasc Imaging Rep.* (2014) 7:9248. 10.1007/s12410-013-9248-7 24688628 PMC3968806

[21] ClinicalTrials.gov. *Cardiotoxicity*(0000) *of Radiation Therapy (CTRT).* (2016) Available online at: https://ClinicalTrials.gov/show/NCT02769299 (accessed January 9, 2019).

[22] Kawel-Boehm N Maceira A Valsangiacomo-Buechel ER Vogel-Claussen J Turkbey EB Williams R Normal values for cardiovascular magnetic resonance in adults and children. *J Cardiovasc Magn Reson.* (2015) 17:29. 10.1186/s12968-015-0111-7 25928314 PMC4403942

[23] Ferreira VM Schulz-Menger J Holmvang G Kramer CM Carbone I Sechtem U Cardiovascular magnetic resonance in nonischemic myocardial inflammation: expert recommendations. *J Am Coll Cardiol.* (2018) 72:3158–76. 10.1016/j.jacc.2018.09.072 30545455

[24] Henriksen PA. Anthracycline cardiotoxicity: an update on mechanisms, monitoring and prevention. *Heart.* (2018) 104:971–7. 10.1136/heartjnl-2017-312103 29217634

[25] Zhang S Liu X Bawa-Khalfe T Lu LS Lyu YL Liu LF Identification of the molecular basis of doxorubicin-induced cardiotoxicity. *Nat Med.* (2012) 18:1639–42. 10.1038/nm.2919 23104132

[26] Neilan TG Coelho-Filho OR Shah RV Feng JH Pena-Herrera D Mandry D Myocardial extracellular volume by cardiac magnetic resonance imaging in patients treated with anthracycline-based chemotherapy. *Am J Cardiol.* (2013) 111:717–22. 10.1016/j.amjcard.2012.11.022 23228924 PMC3578020

[27] Jordan JH Vasu S Morgan TM D’Agostino RB Jr Meléndez GC Hamilton CA Anthracycline-associated T1 mapping characteristics are elevated independent of the presence of cardiovascular comorbidities in cancer survivors. *Circ Cardiovasc Imaging.* (2016) 9:e004325. 10.1161/CIRCIMAGING.115.004325 27502058 PMC5508215

[28] Meléndez GC Jordan JH D’Agostino RB Jr Vasu S Hamilton CA Hundley WG. Progressive 3-month increase in LV myocardial ECV after anthracycline-based chemotherapy. *JACC Cardiovasc Imaging.* (2017) 10:708–9. 10.1016/j.jcmg.2016.06.006 27544895 PMC7890530

[29] Muehlberg F Funk S Zange L von Knobelsdorff-Brenkenhoff F Blaszczyk E Schulz A Native myocardial T1 time can predict development of subsequent anthracycline-induced cardiomyopathy. *ESC Heart Fail.* (2018) 5:620–9. 10.1002/ehf2.12277 29673122 PMC6073029

[30] Galán-Arriola C Lobo M Vílchez-Tschischke JP López GJ de Molina-Iracheta A Pérez-Martínez C Serial magnetic resonance imaging to identify early stages of anthracycline-induced cardiotoxicity. *J Am Coll Cardiol.* (2019) 73:779–91. 10.1016/j.jacc.2018.11.046 30784671

[31] Takagi H Ota H Umezawa R Kimura T Kadoya N Higuchi S Left ventricular T1 mapping during chemotherapy-radiation therapy: serial assessment of participants with esophageal cancer. *Radiology.* (2018) 289:347–54. 10.1148/radiol.2018172076 29989523

[32] Ricco A Slade A Canada JM Grizzard J Dana F Rezai Gharai L Cardiac MRI utilizing late gadolinium enhancement (LGE) and T1 mapping in the detection of radiation induced heart disease. *Cardiooncology.* (2020) 6:6. 10.1186/s40959-020-00061-z 32626602 PMC7329507

[33] Umezawa R Ota H Takanami K Ichinose A Matsushita H Saito H MRI findings of radiation-induced myocardial damage in patients with oesophageal cancer. *Clin Radiol.* (2014) 69:1273–9. 10.1016/j.crad.2014.08.010 25246336

[34] Tuohinen S Skytta T Virtanen V Nikus K Kellokumpu-Lehtinen PL Raatikainen P 30 Radiotherapy-induced changes in breast cancer patients in extra cellular volume and T1 mapping in cardiac magnetic resonance imaging and in ECG six years after radiotherapy treatment. *Eur Heart J Cardiovasc Imaging.* (2019) 20:jez111.008. 10.1093/ehjci/jez111.008

